# On Modern Progress in Ophthalmic Medicine and Surgery

**Published:** 1895-08-10

**Authors:** Robert Brudenell Carter

**Affiliations:** Consulting Ophthalmic Surgeon to St. George's Hospital


### On Modern Progress in Ophthalmic Medicine and Surgery.

By Robert Brudenell Carter, F.R.C.S., Consulting Ophthalmic Surgeon to St. George's Hospital.

### DISEASES OF THE CORNEA.

Conical Cornea (*Continued from page* 265.)

The increased attention which was soon paid to all ocular conditions, as one consequence of the invention of the ophthalmoscope, led in time to the observation that the healing of a corneal ulcer was always or nearly always attended by more or less general flattening of the convexity of the membrane; and the principle of treatment which suggested itself to von Graefe was that this cicatricial flattening might be utilised to counteract the undue bulging of conicity. Von Graefe's original method of procedure was to shave off a thin lamina of sufficient extent from the apex of the cone, to cauterise the resulting solution of continuity until a sufficient ulcer was established, and then to place the eye under the most favourable conditions for healing. The object was to establish a firm central leucoma, composed of tissue more resisting than that of the cornea itself, and therefore calculated to arrest the further progress of the protrusion; while the contraction incidental to the reparative process, or that subsequently occurring in the scar, might serve to restore the portions beyond its boundaries to something like their natural curvature. In favourable cases, the conclusion of this stage of the treatment left the eye with an opaque central cicatrix, which, if sufficiently deep and extensive to render the centre of the cornea unyielding, and to correct the outlines of its lateral parts, was at the same time an absolute impediment to central vision, and a conspicuous disfigurement to the patient. The first of these results was corrected by the subsequent formation of an artificial pupil; the second was concealed by tattooing the scar tissue with Indian ink.

Von Graefe's suggestion was made at a time when cases of conicity had become almost the despair of ophthalmic surgeons, and when it was easy to find a considerable accumulation of persons requiring treatment. The results, both in his own hands and those of others, were at first very uncertain, until experience had determined the proportion which the artificial ulcer should bear to the extent of the malformation, and until many other points of practice had been cleared up. On account of the transparency of the corneal tissue, it was often extremely difficult to shave off a portion of its surface without penetrating into the anterior chamber, and, as instances in which accidental penetration had occurred were often found to yield good results, designed penetration was employed by many surgeons. Some made a puncture and counterpuncture with a fine cataract knife, cut out in a direction somewhat upwards or downwards, and then, seizing the edge of the flap, finished this part of the operation by excising an elliptical piece with scissors. The edges of the aperture would fall together if it were not too large, and only a linear scar would be left. Some carried a suture through the anterior chamber, with a sharply curved needle, before excising the elliptical piece, and then closed the wound by means of the thread. In both these proceedings accidental wounds of the lens, with consequent traumatic cataract, was not unknown, while in more than one instance the lens was expelled through the corneal wound by muscular action. When no disaster occurred, and when the lips of the corneal wound united kindly, the line of union afforded less security against continued expansion than a cicatrix of greater superficial area. On the other hand, the effect of cauterising the wounded cornea was sometimes more extensive than the operator either intended or desired, and left behind so large an opacity that no subsequent lateral pupil could be made to afford useful vision.

Perhaps the most important step towards securing the quality of result which is now constantly obtained, was that made by the late Sir William Bowman, who suggested that the central portion of the cornea might be removed, to whatever depth the surgeon desired, by means of a trephine, which would at the same time limit and define the area of the wound, and permit it to be precisely adjusted to the requirements of each particular case. Such trephines are now commonly made in three sizes, and are furnished with a central stop or plug which limits the depth to which the cutting edge can penetrate, and which can be set to a scale graduated in half millimetres. As a rule, it is not desirable to penetrate the anterior chamber.

In proceeding to operate, the trephine selected should first be perfectly sterilised by boiling in water, and the eye brought fully under the influence of cocaine, to which sufficient atropine should be added to produce large dilatation of the pupil. The lid edges, and the whole of the conjunctival surface, should be well washed with an antiseptic solution of an unirritating character, and the lids held apart by a speculum. Having first set the trephine to the required depth, the surgeon seizes the eye near the corneal margin with fixation forceps, and applies the trephine perpendicularly in such a position that its circle includes the apex of the cone, which does not always coincide with the centre of the cornea. The eye being kept steady by the forceps, the edge of the trephine is firmly pressed against the cornea, while a rotatory motion is imparted to the instrument by the finger and thumb in such a way that it cuts a circular groove into the tissue. The edge of the piece included within the circle is seized with forceps and peeled off. The point of a fine stick of nitrate of silver is next brought into momentary contact with the circle of exposed corneal tissue, which should instantly be touched by a morsel of sterilised wool moistened with a solution of common salt. The surgeon should hold the caustic between his thumb and index finger, and the wool between the middle and ring fingers of the same hand, so that no time may be lost before neutralising the caustic, which otherwise, or if its contact with the surface is more than momentary, will be liable to "run," and to affect a larger surface than is desired. Anything like pressure with the caustic must be carefully avoided, and nothing more than the slightest and most rapidly broken contact is permissible. The lids should then be closed, and covered by an antiseptic dressing, which need not be disturbed for a period of about forty-eight hours. After this time the dressing should be removed daily, and a drop of atropine applied, until cicatrisation is complete. The time required for this purpose will vary with the size of the trephine, and with the healing powers of the patient.

